# Editorial: Neuroprotective and therapeutic effects of progesterone, allopregnanolone, and their derivatives

**DOI:** 10.3389/fendo.2026.1771389

**Published:** 2026-01-15

**Authors:** Rachida Guennoun, Iqbal Sayeed, Donald Stein

**Affiliations:** 1 U1195 Inserm and University Paris-Saclay, Le Kremlin-Bicêtre, France; 2 Department of Emergency Medicine, Emory University, Atlanta, GA, United States; 3 Emeritus College, Emory University, Atlanta, GA, United States

**Keywords:** allopregnanolone, Alzheimer’s disease, membrane progesterone receptor, mitochondrial respiration, neuroprotection, neurosteroids, Parkinson’s disease, stroke

Beyond their classical reproductive roles, steroids exert profound effects on the nervous system (1–3). In particular, progesterone, allopregnanolone, and synthetic progestins have emerged as key players in modulating neuroprotection, synaptic plasticity, mood, stress, cognition and CNS repair (4–7). Extensive preclinical research demonstrated their therapeutic potential, but translating preclinical findings into clinical practice has been elusive.

Allopregnanolone can promote inhibitory neurotransmission and exert rapid antidepressant effects. It is now approved for clinical treatment of postpartum depression (8). Clinical trials of progesterone and allopregnanolone for treating neurodegenerative disease and traumatic brain injury (TBI) had mixed outcomes. Allopregnanolone was evaluated in Phase 1b/2a clinical trials for Alzheimer’s disease (AD), examining its safety and pharmacokinetics. Neuroimaging techniques provided evidence that allopregnanolone may be helpful in promoting regenerative and therapeutic effects for mild AD (9).

Progesterone was evaluated in Phase 2 studies of moderate-to-severe TBI, with early signals of benefit (10, 11); however, two large Phase 3 randomized controlled trials failed to demonstrate its efficacy (12, 13). Subsequent analyses have identified several factors that may have contributed to the failure of these trials, including the high doses of progesterone used; the lack of patient stratification; insufficient consideration of sex- and age-related differences in hormonal status and outcomes; the presence of pre-existing comorbidities; and the use of subjective outcome measures that do not accurately capture deficits or long-term quantitative recovery (5, 14–16). Importantly, while these reviews highlight major translational challenges, they emphasize the pleiotropic potential of progesterone -including anti-inflammatory, anti-apoptotic, neurogenic, and remyelinating effects- in treating complex pathologies such as TBI.

Overall, progesterone and allopregnanolone continue to attract interest as potential therapeutic steroids for neuroprotection and regeneration. Carefully designed future clinical studies, with optimized designs, nuanced patient stratification, and appropriate outcome measures, may help further elucidate their potential role in this evolving field.

The current Research Topic assembles original research articles and a review that highlight mechanistic insights, receptor-specific signaling pathways, and translational opportunities for progesterone and allopregnanolone as neuroprotective agents.

## Allopregnanolone: a multifaceted neurosteroid mediator

Wang et al. provide compelling evidence that calcium signaling represents a unifying mechanism for the pleiotropic effects of allopregnanolone in neurons and astrocytes. Using transcriptomic, mitochondrial, and pharmacological analyses, the authors demonstrate that allopregnanolone enhances synaptogenesis, mitochondrial respiration, and oxidative stress resilience through GABA_A_ and L-type Ca^2+^ channel–dependent pathways. The activation of Ca^2+^/CREB and NRF1-TFAM signaling cascades supports mitochondrial biogenesis and cellular recovery. This comprehensive analysis provides an integrated view of how allopregnanolone coordinates cellular and metabolic processes that may support brain repair and functional recovery; highlighting its promise as a multifaceted neuroprotective agent.

## Progesterone and receptor-specific neuroprotection

Extending this perspective to a disease-specific context, Castelnovo and Thomas explore the neuroprotective role of progesterone in a Parkinson’s disease (PD) cell model. While progesterone has long been studied for its classical genomic effects via nuclear receptors, the authors uncover a critical role of the membrane progesterone receptor α (mPRα/PAQR7) in neuroprotection. Using a human cell-line (SH-SY5Y) as a PD model, they report that progesterone exerts its neuroprotective effects through mPRα/PAQR7 by activating PI3K-AKT and MAPK signaling cascades, which are key pathways for promoting neuronal survival. Nuclear PR and GABA_A_ receptor agonists failed to reproduce this effect, highlighting the specificity of mPRα-mediated signaling. This study underscores the relevance of non-classical progesterone signaling mechanisms in neuroprotection and identifies mPRα as a promising molecular target for developing new therapeutic strategies for PD and related neurodegenerative conditions.

## Neurotrophins, glia, and mechanistic diversity of progesterone signaling

Complementing the other papers, Singh et al. provide an integrative review linking progesterone to brain-derived neurotrophic factor (BDNF) and related signaling networks that contribute to neuroprotection. Their synthesis highlights the importance of glial cells and microRNAs in modulating progesterone’s neuroprotective actions. Importantly, they highlight that the type of progestin—whether natural or synthetic—can profoundly influence neuroprotective outcomes, with significant implications for clinical translation and hormone therapy design.

## Steroid hormone biomarkers and stroke risk prediction

The translational aspect of this Research Topic is provided by Yang et al., who propose a biomarker-based approach to predict stroke risk using hair-derived hypothalamic-pituitary-gonadal axis hormone levels. Their findings suggest that decreased, pre-stroke testosterone and progesterone concentrations in hair can serve as predictive indicators of stroke vulnerability. These preliminary findings introduce a potential non-invasive biomarker approach offering new possibilities for early cerebrovascular risk assessment; thus, expanding the clinical relevance of neuroendocrine markers in preventive neurology.

## Hormonal dysregulation and neurodegenerative vulnerability

Yang et al. explore shared neuroendocrine mechanisms linking premenstrual dysphoric disorder (PMDD) and Alzheimer’s disease (AD). They identify overlapping pathways involving allopregnanolone fluctuations, GABAergic and serotonergic dysregulation and the APOE-ϵ4 allele. This conceptual framework can be taken to suggest that PMDD may be a biological precursor state for AD, emphasizing the role of sex hormones in late-life neurodegenerative risk. Their analysis supports the view that hormonal instability may contribute to long-term neurodegenerative risk in women while emphasizing the need to incorporate sex-specific endocrine dynamics into models of AD pathogenesis and early intervention strategies.

## Integrative perspectives and future directions

Collectively, the articles in this Research Topic bring together recent advances in the mechanistic, cellular, and translational dimensions of neurosteroid research. The papers highlight emerging molecular targets such as calcium signaling pathways, mitochondrial dynamics, and membrane progesterone receptors and propose innovative translational and predictive approaches. These contributions highlight the multi-level complexity of neurosteroid action from molecular signaling to systems physiology and development of clinical biomarkers. Taken together, these contributions reinforce several principles:

1. Neurosteroid actions are inherently pleiotropic, involving coordination between neurotransmitter systems, mitochondrial function and signaling pathways.
2. Receptor specificity matters: the differential effects of nuclear versus membrane progesterone receptors underscore the need for precise pharmacological targeting.
3. Sex and age are critical biological variables that shape neurosteroid efficacy, influencing outcomes in preclinical studies as well as for designing clinical trials.
4. Translational biomarkers, such as plasma or hair-based sex steroid hormone measures, may serve as valuable tools for risk stratification and therapeutic monitoring.

## Conclusion

Progesterone and allopregnanolone and their derivative continue to emerge as promising therapeutic steroids for neuroprotection and regeneration. Additional clinical investigations, designed with refined methodologies, thoughtful patient stratification, and clinically meaningful outcome measures, would be valuable to further clarify and confirm their clinical potential.
